# Effects of qiming granule for diabetic macular edema: A protocol for systematic review and meta-analysis

**DOI:** 10.1097/MD.0000000000017496

**Published:** 2019-10-18

**Authors:** Zhipeng Hu, Maoyi Yang, Chunguang Xie, Hong Gao, Xiaoxu Fu, Hongyan Xie, Ya Liu

**Affiliations:** Hospital of Chengdu University of Traditional Chinese Medicine, PR China.

**Keywords:** diabetic macular edema, protocol, qiming granule, systematic review and meta-analysis

## Abstract

**Background:**

Diabetic macular edema is a further complication of diabetes. It is an important type of diabetic eye disease and the main cause of blindness of diabetic patients. Qiming granule is a Chinese patent medicine widely used in the treatment of diabetic macular edema. There are some reports about this medicine for macular edema. At present, there is only 1 systematic review on qiming granule in the treatment of diabetic macular edema. However, there are many defects in this article, so it is necessary to re-summarize and evaluate the existing evidence.

**Methods and analysis:**

Three English database and 4 Chinese databases other sources will be searched. Two methodological trained researchers will read the title, abstract and full texts, and independently select the qualified literature according to inclusion and exclusion criteria. After assessment of the risk of bias and data extraction, we will conduct meta-analyses for outcomes including central macular thickness, optimum corrected vision, overall effect rates, and adverse effects. The heterogeneity of data will be investigated by Cochrane *χ*^2^ and *I*^*2*^ tests. We build 3 hypotheses for subgroup analysis according to the guidance for a credible subgroup effect: Disease status at baseline, duration of intervention, type of concomitant medication. Sensitivity analysis will be conducted to evaluate the stability of the results. Then publication bias assessment will be conducted by funnel plot analysis and Egger test. Finally, we will use the Grading of Recommendations Assessment, Development and Evaluate system to evaluate the quality of evidence.

**Results:**

The results of our research will be published in a peer-reviewed journal.

**Conclusion:**

In our study, the evidence of qiming granule in the treatment of macular edema was comprehensively summarized and carefully evaluated. It will provide more options for clinical treatment of the disease.

**PROSPERO registration number:**

CRD42018108626.

## 1 Introduction

Diabetes mellitus (DM) is a metabolic disease characterized by persistent hyperglycemia.^[1]^ Many complications can occur in the later stage of DM, including diabetic eye disease (DED).^[2]^ Diabetic macular edema (DME) is an important type of DED. It is characterized by a swelling of the macular caused by ischemia; this can lead to blood vessel leakage and changes in permeability, eventually destruction of the blood-retinal barrier.^[3]^ According to the 8th edition of the IDF Diabetes Atlas 2017, the prevalence of DME in the America region was 6.4%, 6.3% in the South–East Asia region and 8.9% in Europe.^[4–9]^ Treatment of DME puts a heavy burden on the government's finances.^[10–12]^

The main treatments of DME include glucocorticoids, laser treatment, and anti-vascular endothelial growth factor (VEGF) agents.^[13]^ These treatments have its own limitations. Glucocorticoid therapy can effectively improve the visual acuity of DME patients but may increase the incidence of cataract and increase intraocular pressure.^[14]^ The laser treatment can cause trauma to the eyes, such as destruction of photoreceptors, gradual expansion of laser spots, induction of choroid neovascularization, subretinal fibrosis, etc.^[15,16]^ Clinical trials have shown that intraocular injections of anti-VEGF drugs are effective in treating DME.^[17,18]^ Although intravitreal anti-VEGF therapy is safe, the treatment of DME requires multiple injections, which has a massive local impact on the retina, and its complications increase correspondingly during the whole treatment process. Complications include infectious endophthalmitis, retinal detachment, elevated intraocular pressure or glaucoma. So new therapies are needed.

Qiming granule (QG) is the first Chinese patent medicine approved by the China Food and Drug Administration (CFDA) for the treatment of DED (approved No. Z20090036). It is composed of astragalus, pueraria, rehmannia, *Fructus lycii*, motherwort, puhuang and leech. Experimental studies have shown that it can exert a good effect on DME through a variety of ways.^[19–22]^ It has become the most widely used medicine for treating DME in clinical practice since approved and a series of clinical studies have been carried out about it. In Chinese diabetes prevention guidelines published in 2017, QG was mentioned as an alternative treatment for DED.^[23]^ However, the guideline did not grade the evidence. There is only 1 meta-analysis about QG in the treatment of diabetic macular edema, and there are many defects in this study.^[24]^ Firstly, the author did not carry out a comprehensive literature search, which led to the omission of some original studies in the systematic evaluation and some documents that should not be included meta-analysis were included. Secondly, in data analysis, the author failed to merge data correctly and reasonably, and also, the author did not carry out evidence classification, which limited the guiding significance of its research. Therefore, a high-quality research is urgently needed. In this study, we will systematically collect clinical studies of qiming granule on the treatment of DME and conduct systematic review and meta-analysis to provide reliable evidence for clinical practice.

## 2 Methods and analysis

### 2.1 Study registration

A prospective protocol including the detailed search strategy and methods of data analysis have been prepared according to the Preferred Reporting Items for Systematic Reviews and Meta-analysis (PRISMA) of Observation Studies in Epidemiology recommendations for study reporting. This systematic review and meta-analysis protocol is reported according to the Preferred Reporting Items for Systematic Reviews and Meta-analysis Protocols (PRISMA-P) checklist.^[25]^

### 2.2 Inclusion criteria

Those randomized controlled trials that treated diabetic macular edema with qiming granule in combination with placebo or conventional treatments will be included in our study. Conventional treatments include medical therapy like anti-VEGF agents such as bevacizumab, ranibizumab, and aflibercept. Focal laser photocoagulation and Intravitreal glucocorticoids are also used for diabetic macular edema. The diagnosis of diabetic macular edema was based on the results of stereoscopic viewing, fluoresce in angiography, and optical coherence tomography (OCT; a noninvasive, low-energy laser imaging technology). The diagnosis is according to Guidelines for the prevention and treatment of type 2 diabetes in China (2007). In order to obtain as many references as possible and reduce bias, there will be no limitation about sex, ages, and disease severity and other factors.

Clinically, diabetic macular edema usually occurs in conjunction with non-proliferative diabetic retinopathy or proliferative retinopathy. So, in our researches, those studies which used “diabetic retinopathy” as the title but mentioned macular edema as outcomes will also be included for full-article assessing.

### 2.3 Exclusion criteria

Studies that met the following criteria will be excluded for the meta-analysis:

1. macular edema caused by other reasons other than diabetes such as macular edema after ultra-phacoemulsification for cataract or caused by obstruction of the central retinal vein;
2. in some studies, the authors uses QG and other medicines together in experimental group, since qiming granule is not the main intervention in these studies, these studies will be excluded;
3. studies whose outcomes are not directly related to DME;
4. repeatedly published articles;
5. Abstract articles, conference papers, review articles, systematic review and meta-analysis will be excluded because there no relevant data that could be used in our research;
6. studies that are not randomized controlled trials will be excluded.

### 2.4 Outcomes

#### 2.4.1 Primary outcomes

Central macular thickness. This is examined by optical coherence tomography.

#### 2.4.2 Secondary outcomes

1. Optimum corrected vision. This is conducted by using a standard visual acuity chart;
2. Total effective rate; the judgment of effectiveness takes into account both the improvement of examination indicators and the alleviation of clinical symptoms.
3. Time and frequency of recurrence of macular edema.
4. Any adverse effects in the whole process of taking medicine.

### 2.5 Study search

We will search 3 English database including PubMed, Embase, Cochrane Library Central Register of Controlled Trials and 4 Chinese databases including China National Knowledge Infrastructure (CNKI) database, Wanfang Data Knowledge Service Platform, the VIP information resource integration service platform (cqvip), China Biology Medicine Disc (Sino Med) with a language limitation of English and Chinese. In addition, we will also search Google scholar, Baidu Scholar to find out unpublished researches or other related literature. And above all, the Chinese Clinical Trial Registry (ChiCTR) and ClinicalTrials.gov will also be searched. A manual search will be conducted at the library of Chengdu University of Traditional Chinese Medicine.

A search strategy that combines MeSH terms and free words will be adopted by us. The search terms used will be as follows: “qiming granule”, “qiming”, “qiming keli”, “Diabetic retinopathy”, “Diabetic Retinopathies”, “Retinopathies, Diabetic”, “Retinopathy, Diabetic”, “Diabetic macular edema”, “Macular Edema”, “Edema, Macular”, “Irvine-Gass Syndrome”, “Irvine Gass Syndrome”, “Syndrome, Irvine-Gass”, “Cystoid Macular Edema, Postoperative”, “Macular Edema, Cystoid”, “Edema, Cystoid Macular”, “Cystoid Macular Dystrophy”, “Macular Dystrophy, Dominant Cystoid”, “Central Retinal Edema, Cystoid”, “Cystoid Macular Edema”. Two authors (Zhipeng Hu and Maoyi Yang) will search and screen all the citations independently. The process of search is presented in the following Table 1.

**Table 1:**
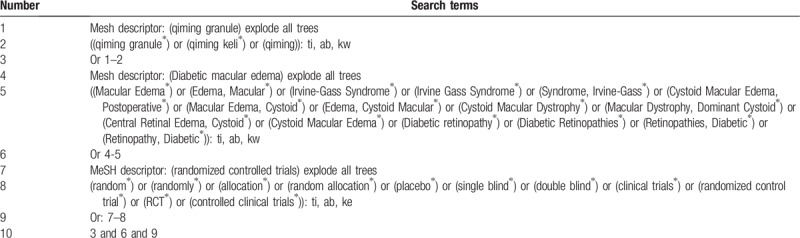
Example of Cochrane search strategy.

### 2.6 Study selection

**Figure 1:**
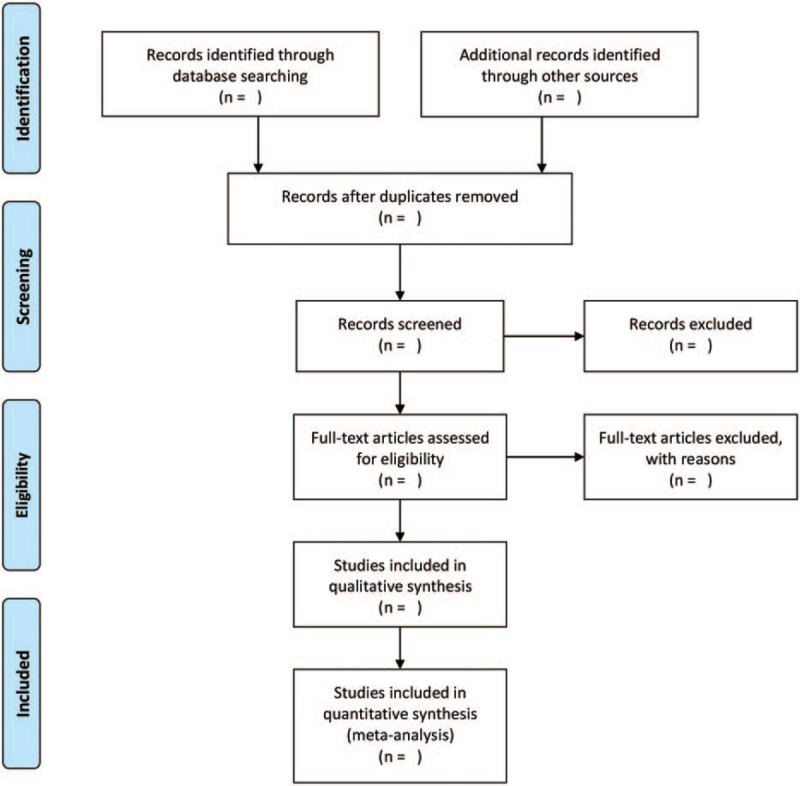
Flow chart of study selection.

We will manage the electronic citations we downloaded from the above databases in Endnote X8 for Mac (Thomson Reuters, USA). And then 2 methodological trained researchers will read the titles and abstracts of citations and screen the citations according to the inclusion and exclusion criteria. Those articles that meet the criteria will be further determined for inclusion by reading the full text. A final decision will be made through consensus when there were discrepancies. A flow chart will be drawn to show the process of study selection (Fig. 1).

### 2.7 Data extraction

We will extract the data of those qualified articles into Microsoft Excel. For each study, the following data will be extracted: The first authors of the article, year of publication, interventions in experimental group, interventions in control group, time of treatment, course of disease, number of patients in each group, ages and sex of patients, outcomes and safety data. If there is not enough data in a study, we will contact the corresponding author for more detailed data. If the methodological details are not told in papers, we will contact for more explanation.

### 2.8 Risk of bias assessment

Two reviewers will assess the risk of bias of included articles by using the Cochrane collaboration tool. This is an established and reliable tool for assessing the risk of bias in studies. In this tool, the risk of bias of a trial is assessed through 7 items: random sequence generation (selection bias), allocation concealment (selection bias), blinding of participants and personnel (performance bias), blinding of outcome assessment (detection bias), incomplete outcome data (attrition bias), selective reporting (reporting bias), other bias. Each item is classified as “Low risk”, “High risk” or “Unclear risk”.^[26]^ Two reviewers will conduct the risk of bias assessment independently and any disagreements will be solved by a discussion of all reviewers.

### 2.9 Data analysis

Endnote X8 for Mac (Thomson Reuters, USA) will be used to manage our citations, and Review Manager Version 5.3 and stata 14.0 software for Mac will be used to create forest plots and conduct subgroup analysis and sensitivity analysis. For the binary variable, the effect size will be represented as risk ratio (RR) and 95% confidence interval (CI) and a Mantel–Haenszel (M–H) method will be used to calculate them. For continuous variable, the effect size will be represented as mean difference (MD) and 95% CI. If 1 studies report its standard error (SEM) other than standard deviation (SD), we will convert SEM into SD. The heterogeneity of data will be investigated by Cochrane χ^2^ and *I*^*2*^ tests.^[27]^ The statistical heterogeneity is considered substantial when *P* < .05 and *I*^2^ > 50%. If *P* > .05 and *I*^2^ < 50%, then the studies included is homogeneous and the differences between them can be ignored. If there is significant heterogeneity, the random effect model will used to pool data, and if there is no significant heterogeneity, then the fixed effect model will be used. If quantitative synthesis is not appropriate due to substantial heterogeneity, then the results will be presented with tables and figures.

### 2.10 Investigation of heterogeneity

If there is substantial heterogeneity between studies, then we will conduct subgroup analysis and meta-regression to explore the heterogeneity. We build 3 hypotheses for subgroup analysis: Disease status at baseline, duration of intervention, type of concomitant medication.^[28]^ Since there is no significant correlation between the onset of DME and the course of DM, the age of patients and the course of DM are not one of the hypotheses of subgroup analysis.^[3]^ We will conduct our subgroup analysis according to these subgroup hypotheses. Then we will evaluate the credibility of our subgroup analysis according to the guidance for credible subgroup analysis.^[29]^ If there are enough studies, then meta regression will be conducted to further explore the sources of heterogeneity.

### 2.11 Sensitivity analysis

In order to investigate the stability of the results, we will conduct a sensitivity analysis for the outcomes. We will exclude each study that is included in the analysis 1 by 1, and then re-analyze and pooled the data and compare the difference between the re-obtained effects and the original effects. In this way, we will be able to assess the impact of individual studies on the overall results and whether the results are robust.

### 2.12 Publication bias assessment

If there are more than ten studies included, a funnel plot analysis will be drawn to assess the publication bias and Egger test will be conducted for statistical investigation.^[30,31]^ The publication bias is considered to exist if *P* < .05.

### 2.13 Summary of finding tables

At last, the summary of finding tables for each outcome will be generated by Grading of Recommendations Assessment, Development and Evaluate system (GRADE). This is a widely used tool in evaluating the quality of assessment.^[32]^ In this table, the evidence will be shown from 5 domains: certainty assessment, number of patients, effect, certainty, and importance. In the GRADE system, the quality of evidence can be defined as “high”, “moderate”, “low”, and “very low”.

### 2.14 Patient and public involvement

Patient and public were not involved in this study.

### 2.15 Ethics and dissemination

Ethical approval is not needed for this meta-analysis. Our study comprehensively evaluates the existing research evidence of qiming granule and is bound to provide evidence-based medical support for clinical workers. The results of our research will be published at a peer reviewed journal.

## 3 Discussion

DME is an important form of diabetic eye disease and the leading cause of blindness of diabetic patients. QG has become the most widely used medicine for treating DME in clinical practice since approved and a series of clinical studies have been carried out about it. However, at present, there is no systematic and comprehensive summary of the existing clinical evidence, which limits the clinical application of QG. In this study, we will conduct this systematic review and meta-analysis to provide more evidence-based medical support for clinical use of QG.

In this study, in order to draw more accurate and reliable conclusions, we will use the following analytical methods in the research process. We will conduct subgroup analysis and regression analysis to explore the possible heterogeneity between studies. To avoid meaningless post-analysis, we will conduct our subgroup analysis according to preset subgroup hypotheses. And then we will assess the subgroup analysis according to the criteria of reliability of subgroup analysis. Finally, we will classify the existing evidence to provide a better guide for clinical use.

## Author contributions

**Conceptualization:** Zhipeng Hu, Maoyi Yang, Chunguang Xie.

**Data curation:** Hong Gao, Xiaoxu Fu.

**Formal analysis:** Maoyi Yang, Zhipeng Hu.

**Funding acquisition:** Chunguang Xie.

**Investigation:** Hongyan Xie, Ya Liu.

**Methodology:** Zhipeng Hu, Maoyi Yang, Chunguang Xie.

**Project administration:** Chunguang Xie.

**Resources:** Zhipeng Hu, Chunguang Xie.

**Software:** Zhipeng Hu, Maoyi Yang.

**Supervision:** Hongyan Xie.

**Writing - original draft:** Zhipeng Hu.

**Writing – review and editing:** Chunguang Xie and Ya Liu.

Zhipeng Hu orcid: 0000-0003-1524-6452.
